# Combination of Selective Immunoassays and Mass Spectrometry to Characterize Preproghrelin-Derived Peptides in Mouse Tissues

**DOI:** 10.3389/fnins.2017.00211

**Published:** 2017-04-20

**Authors:** Rim Hassouna, Dominique Grouselle, Giovanni Chiappetta, Joanna Lipecka, Oriane Fiquet, Catherine Tomasetto, Joëlle Vinh, Jacques Epelbaum, Virginie Tolle

**Affiliations:** ^1^Centre de Psychiatrie et Neurosciences, UMR-S 894 Institut National de la Santé et de la Recherche Médicale, Université Paris Descartes, Sorbonne Paris CitéParis, France; ^2^Department of Pediatrics, Naomi Berrie Diabetes Center, Columbia University Medical CenterNew York, NY, USA; ^3^ESPCI Paris, PSL Research University, Spectrométrie de Masse Biologique et Protéomique (SMPB), CNRS USR 3149Paris, France; ^4^UMR-7104 Centre Nationnal de la Recherche Scientifique/U596, Institut National de la Santé et de la Recherche Médicale, Institut de génétique et de biologie moléculaire et cellulaire, Université de StrasbourgIllkirch, France; ^5^UMR 7179 Centre Nationnal de la Recherche Scientifique, MNHN, Adaptive Mechanism and Evolution (MECADEV)Brunoy, France

**Keywords:** acyl ghrelin, desacyl ghrelin, obestatin, immunoreactivity, mass spectrometry

## Abstract

Preproghrelin is a prohormone producing several preproghrelin-derived peptides with structural and functional heterogeneity: acyl ghrelin (AG), desacyl ghrelin (DAG), and obestatin. The absence of selective and reliable assays to measure these peptides simultaneously in biological samples has been a limitation to assess their real proportions in tissues and plasma in physiological and pathological conditions. We aimed at reliably measure the ratio between the different preproghrelin-derived peptides in murine tissues using selective immunoassays combined with a highly sensitive mass spectrometry method. AG-, DAG-, and obestatin-immunopositive fractions from the gastrointestinal tract of adult wild-type and ghrelin-deficient mice were processed for analysis by mass spectrometry (MS) with a Triple Quadrupole mass spectrometer. We found that DAG was predominant in mouse plasma, however it only represented 50% of total ghrelin (AG+DAG) production in the stomach and duodenum. Obestatin plasma levels accounted for about 30% of all circulating preproghrelin-derived peptides, however, it represented <1% of total preproghrelin-derived peptides production (AG+DAG+Obestatin) in the stomach. Assays were validated in ghrelin-deficient mice since neither ghrelin nor obestatin immunoreactivities were detected in their stomach, duodenum nor plasma. MS analyses confirmed that obestatin-immunoreactivity in stomach corresponded to the C-terminal amidated form of the peptide but not to des(1–10)-obestatin, nor to obestatin-Gly. In conclusion, specificity of ghrelin and obestatin immunoreactivities in gastrointestinal tissues using selective immunoassays was validated by MS. Obestatin was less abundant than AG or DAG in these tissues. Whether this is due to inefficient processing rate of preproghrelin into mature obestatin in gastrointestinal mouse tissues remains elusive.

## Introduction

Preproghrelin is a complex prohormone that, upon post-translational processing, leads to the production of several derived peptides with structural and functional heterogeneity. Ghrelin is a 28 amino acid peptide originating from the stomach (Kojima et al., 1999; Hosoda et al., 2000; Tomasetto et al., 2000) and identified as the endogenous ligand of the Growth Hormone Secretagogue Receptor (GHS-R; Howard et al., 1996). The addition of an acyl-group by the Ghrelin-O-Acyl-Transferase (GOAT; Yang et al., 2008), enables ghrelin (Acyl-ghrelin, AG) to stimulate GH secretion and appetite (Tolle et al., 2001, 2002). Another endogenous form of ghrelin is desacyl ghrelin (DAG) reported as the most abundant form in plasma (Hosoda et al., 2000). Its specific roles are to regulate glucose, lipid, and bone metabolism (Delhanty et al., 2013). Obestatin is a 23 amino acid amidated peptide derived from the same precursor as ghrelin. Originally isolated as the endogenous ligand for the GPR39 and described as an anorexigenic factor in rodents (McKee et al., 1997; Zhang et al., 2005), its physiological relevance has since been questioned (Zhang et al., 2005; Bresciani et al., 2006; Lauwers et al., 2006; Seoane et al., 2006; Yamamoto et al., 2007). Both DAG and obestatin interact pharmacologically with AG to modulate food intake, GH secretion or glucose metabolism through yet unidentified receptors (Hassouna et al., 2012; Delhanty et al., 2013; Gargantini et al., 2013). Previous studies found equimolar ratios of plasma AG and obestatin levels in the rat (Zhang et al., 2005; Zizzari et al., 2007), consistent with both peptides being processed from the same prohormone. However, evidence that tissue specific splicing variant encoding obestatin but not ghrelin exist in humans suggests that obestatin could also be produced independently of ghrelin (Seim et al., 2009).

With regard to the literature, many issues remain to be addressed concerning obestatin: its main source of production in the body and its abundance relative to ghrelin as well as its molecular form and way of processing. Obestatin was initially extracted from rat stomach and found in rat plasma (Zhang et al., 2005). In addition, obestatin immunoreactivity was detected in a number of human tissues using immunohistochemistry (Grönberg et al., 2008) and in cultured pancreatic islets *in vitro* (Granata et al., 2008). However, other studies failed to detect significant amounts of obestatin in rat plasma or stomach using radioimmunoassay (RIA) coupled to High Performance Liquid Chromatography (HPLC; Bang et al., 2007; Mondal et al., 2008).

The absence of selective and reliable assays to measure all three preproghrelin-derived peptides (AG, DAG, and obestatin) simultaneously in biological samples is an obstacle to further characterization of their specific physiological and pathophysiological functions. In this study, we developed selective immunoassays combined to a highly sensitive targeted mass spectrometry method in order to reliably measure and characterize the ratios of the different preproghrelin-derived peptides in mice. Preproghrelin gene deficient mice that do not produce AG or DAG (Hassouna et al., 2014) were used as negative controls to further validate the immunoreactivity and mass spectrometry assays.

## Materials and methods

### Animals

Dissections were performed on 7–12 weeks old preproghrelin deficient (*ghrl*−/−) mice and wild type (*ghrl*+/+) littermates backcrossed on the C57BL/6J genetic background as previously reported (Hassouna et al., 2014). Mice were housed in a room under controlled illumination (0700–1900 h) and temperature (22–24°C) and had free access to food and water. Offsprings were genotyped by PCR amplification of tail DNA. All experiments were carried out in accordance with the European Communities Council Directive (86/609/EEC) and were approved by the Animal Experimentation Committee of Paris Descartes University (agreement number 03422.02).

### Peptides

Peptides used as standards in liquid chromatography (LC) were provided by NeoMPS (Strasbourg, France): rat/mouse acyl ghrelin (AG) and desacyl ghrelin (DAG), rat/mouse amidated obestatin (obestatin-NH_2_), Des(1–10)-obestatin and obestatin-Gly (Sequences presented in Table S1 and Figure S1).

### Dissection, extraction, and purification of tissue samples

Gastric epithelia, 1 cm proximal duodenum, small intestine and colon were collected and the tissues were extracted in 2N acetic acid during 10 min at 90°C, sonicated and frozen at −80°C for 24 h. The homogenate was centrifuged 20 min at 12,000 g at 4°C. Supernatants were lyophilized and further dissolved in phosphate assay buffer. Extracts were first filtered on a 10 kDa filter (Amicon Ultra, Millipore, Molsheim, France) then purified using SepPak C18 columns (Waters, Saint-Quentin-en-Yvelines, France). Briefly, the supernatants were loaded onto a SepPak C18 cartridge pre-equilibrated in 0.1% trifluoroacetic acid (TFA). The samples were desalted with aqueous TFA 0.1% and eluted with an acetonitrile gradient (10–100%).

### Plasma collection and processing

Blood samples were collected from trunk blood on EDTA (1 mg/ml) and PHMB (0.4 mM final), a serine protease inhibitor and centrifuged at 1,000 g during 10 min at 4°C. Plasma were immediately acidified with HCl (0.1 M final) and stored at −80°C.

### Hormone assays

Immunoreactivities were measured in plasma, whole tissue and SepPak fractions (10–70 and 100%) with selective sandwich immunoassays for AG, DAG (SPIbio Bertin pharma, A05118 for the acyl form and A05117 for the desacyl form, Montigny-le-Bretonneux, France) and competitive assay for obestatin (Phoenix Pharmaceuticals, Burlingame, USA). Immunoreactive fractions were further analyzed by Mass Spectrometry (MS).

### Mass spectrometry

Mass Spectrometry (MS) analyses were performed in the Selected Reaction Monitoring (SRM) mode using a high pressure nanoLC (U3000 RSLC, Thermo Fisher Scientific) coupled to a triple quadrupole (QqQ) mass spectrometer (TSQ Vantage™, Thermo Fisher Scientific, San Jose, CA, USA) in nano-ESI mode. Briefly, peptides were loaded and desalted on a C18 cartridge (C18 PepMap, 3 mm, 100 A°, 75 mm i.d., 2 cm length) using a loading buffer containing 0.05% aq TFA/acetonitrile 98:2 (v/v) at 10 μL/min. Peptides were then separated on a C18 analytical column (C18 PepMap, 2 mm, 100 A°, 75 mm i.d., 15 cm length) with a 60 min gradient from 99% A [0.1% aq formic acid/acetonitrile 98:2 (v/v)] to 50% B [0.1% aq formic acid/acetonitrile 10:90 (v/v)] at 300 nL/min. Standard was injected before each series of experiments. Blank runs were interposed until necessary to avoid peptide carry-over effects. QqQ parameters were set as follows: first and third quadrupole widths set at 0.7, scan time 200 ms/transition and total dwell time 3 s (method performed in unscheduled mode). The transitions for AG (retention time 29.1 min) were 553.1 (precursor ${\text{MH}}_{6}^{6+}$) → 513.3 (y_4_); 641.4 (y_5_); 712.4;(y_6_); 809.5 (y_7_) 906.5(y_8_). The transitions for DAG (retention time 20.0 min) were 532.1 (precursor ${\text{MH}}_{6}^{6+}$) → 513.3 (y_4_); 641.4 (y_5_); 712.4; (y_6_); 809.5 (y_7_); 906.5(y_8_). The transitions for obestatin-NH_2_ (retention time 38.3 min) were 630.8 (precursor ${\text{MH}}_{5}^{5+}$) → 262.1(b_2_); 416.2 (y_4_); 553.3 (y_5_); 681.4;(y_6_); 972.5 (y_8_). The transitions for obestatin-Gly (retention time 33.4 min) were 858.8 (precursor ${\text{MH}}_{3}^{3+}$) → 262.1 (b_2_); 333.2 (b_3_); 473.2 (y_5_); 610.3 (y_6_) 1029.5(y_9_). The transitions for des(1–10)-obestatin (retention time 17.9 min) were 476.9 (precursor ${\text{MH}}_{3}^{3+}$) → 201.1 (b_2_); 553.2 (y_5_); 681.3;(y_6_); 809.5 (y_7_); 972.5(y_8_) (Supplementary Table S1).

## Results

### Preproghrelin-derived peptides immunoreactivities in the gastrointestinal tract and plasma

In adult mice, stomach is known to be the major source of ghrelin, however the main source of obestatin remains elusive. Accordingly, since ghrelin gene is widely expressed throughout the gastro-intestinal (GI) tract, we thus measured all preproghrelin-derived peptides in the different portions of the GI tract (stomach, duodenum, intestine, and colon) in mice using selective antibodies (Figure S1). Amongst all preproghrelin-derived peptides, AG and DAG were the most abundant forms found in stomach. In this tissue, AG represented 50%, DAG 50%, and obestatin <1% of all preproghrelin-derived peptides. Although their proportions varied in the different sections of the GI tract, ghrelin (AG and DAG) remained predominant compared to obestatin. In duodenum, AG represented 34%, DAG 38%, and obestatin 29% of all preproghrelin-derived peptides. Differences in proportions of obestatin relative to ghrelin may partly be due to the utilization of different techniques to detect specific immunoreactivities and revelation procedures (sandwich enzymoimmunoassays for ghrelin vs. competitive radioimmunoassay for obestatin). Whereas, AG and DAG levels were gradually decreasing from caudal to distal region of the GI tract, obestatin concentrations were slightly higher in the duodenum than in the stomach (Table 1). Both the intestine and colon produced small amounts of all three peptides (data not shown).

**Table 1:** **Tissue content or concentrations, percentage and molar ratio of preproghrelin-derived peptides immunoreactivities in the stomach, duodenum, and plasma of ***ghrl***+/+ mice (***n*** = 6–10)**.

	**Acyl ghrelin (AG)**	**Desacyl ghrelin (DAG)**	**Obestatin**	**AG/TG**	**DAG/TG**	**Obestatin/TG**
**STOMACH**						
Tissue content (pmol/tissue)	184 ± 9	197 ± 19	0.65 ± 0.02		Molar ratio	
% of total preproghrelin-derived peptides	49 ± 3%	51 ± 3%	0.17 ± 0.01%	1/2	1/2	1/616
**DUODENUM**						
Tissue content (pmol/tissue)	1.32 ± 0.39	1.39 ± 0.42	1.00 ± 0.52		Molar ratio	
% of total preproghrelin-derived peptides	34 ± 6%	38 ± 10%	29 ± 12%	1/2	1/2	1/2.5
**PLASMA**						
Concentration (pmol/L)	103 ± 29	370 ± 44	210 ± 79		Molar ratio	
% of total preproghrelin-derived peptides	16 ± 4%	57 ± 5%	27 ± 8%	1/5	1/1.2	1/1.7

*AG, Acyl ghrelin; DAG, Desacyl ghrelin; TG, Total Ghrelin (AG+DAG). Limits of detection are 0.03 pmol for AG and DAG and 0.08 pmol for obestatin. Data are expressed as Mean ± SEM, molar ratio or as percentages of total preproghrelin-derived peptides (AG+DAG+obestatin)*.

Previous studies using competition assays indicated that DAG accounts for 80–90% of total circulating ghrelin (Hosoda et al., 2000). In the present study, DAG accounted for nearly 80% of total circulating ghrelin (i.e., AG+DAG) and 60% of total circulating preproghrelin-derived peptides (i.e., AG+DAG+Obestatin; Table 1). Obestatin plasma levels represented about 30% of all circulating preproghrelin-derived peptides, which is much higher than its proportion in gastrointestinal tissues (Table 1).

### Mass spectrometry (MS) analysis of preproghrelin-derived peptides in *ghrl*+/+ and *ghrl*−/− mice

**Figure 1:**
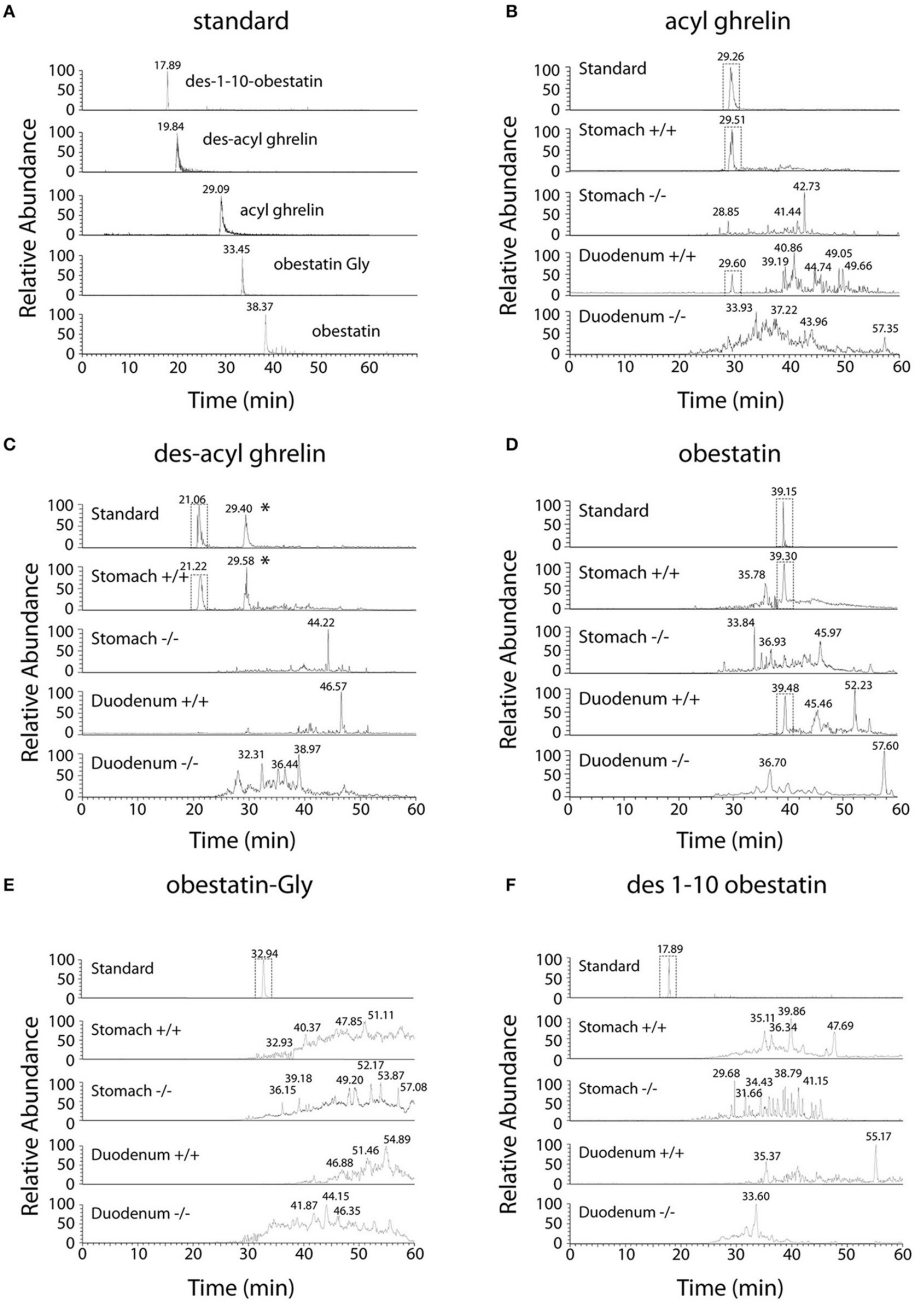
**Chromatographic profiles of SRM analyses related to preproghrelin-derived peptides in the stomach and duodenum from 60% chromatographic fractions in ***ghrl***+/+ and ***ghrl***−/− mice**. SRM analyses of the synthetic peptide (standard) and tissue samples obtained from the fraction eluted with 60% acetonitrile: **(A)** standard, **(B)** acyl ghrelin, **(C)** des-acyl ghrelin, **(D)** obestatin, **(E)** obestatin-Gly, **(F)** des(1–10)-obestatin. Acyl ghrelin and obestatin-NH_2_ were present in both the stomach and duodenum of *ghrl*+/+ mice but absent in the tissues of *ghrl*−/− mice. Des-acyl ghrelin was present in the stomach but not in the duodenum of *ghrl*+/+ mice and absent in the tissues of *ghrl*−/− mice. The star (^*^) denotes a peak in ghrelin chromatogram that is related to the “in source” neutral loss of the acyl group of acyl ghrelin converted partially acyl ghrelin to desacyl ghrelin after LC separation. Obestatin-Gly and des(1–10)-obestatin were absent in both the stomach and duodenum of *ghrl*+/+ and *ghrl*−/− mice. See experimental section for SRM method design.

Selected Reaction Monitoring (SRM) method was set up using synthetic peptides AG, DAG, Obestatin-NH_2_, Des(1–10)-Obestatin and Obestatin-Gly. The SRM method selected the best five transitions obtained by testing all the theoretic “y” and “b” ion fragments from ${\text{MH}}_{3}^{3+}$ to ${\text{MH}}_{6}^{6+}$ precursors (Figure 1). The detection limits for AG and DAG was 10–50 fmol and for obestatin-NH_2_ and its derivatives 1–5 fmol.

As preproghrelin-derived peptides are highly concentrated in stomach and duodenum, purified SepPak fractions from these tissue protein extracts from both *ghrl*+/+ and *ghrl*−/− mice were used for mass spectrometry. Preproghrelin-derived peptides immunoreactivities were retrieved in stomach and duodenum of *ghrl*+/+ mice but not in the same tissues in *ghrl*−/− mice (Table 2 and data not shown). The residual immunoreactivity for obestatin found in two out of six *ghrl*−/− mice was unspecific, since it did not correspond to the correct specific masses in MS experiments (See below). To decrease the dynamic range and the complexity of the whole protein extract, samples were first depleted to conserve only peptides below 10 kDa. The resulting mixtures were further submitted to solid phase extraction on C18 stationary phase to remove hydrophilic species. According to standard synthetic peptides properties, the endogenous peptides of interest were expected to be eluted using 10–70% acetonitrile. The fractions were submitted to stepwise elution with successive 10% increments of acetonitrile. Immunoreactivity of each preproghrelin-derived peptides (AG, DAG, and Obestatin) was measured in each eluted fraction. In *ghrl*+/+ mice, 30, 40, and 60% acetonitrile-containing fractions presented the most intense immunoreactivity and were further analyzed by SRM LC-MS. The matched fractions obtained from *ghrl*−/− mice contained no immunoreactivity for all three preproghrelin-derived peptides and were also analyzed in SRM LC-MS.

**Table 2:** **Preproghrelin-derived peptides immunoreactivities in the stomach and duodenum of ***ghrl***+/+ and ***ghrl***−/− mice**.

	***Ghrl*+/+ (*n* = 6)**		***Ghrl−/−* (*n* = 6)**	
	**Stomach (pg)**	**Duodenum (pg)**	**Stomach (pg)**	**Duodenum (pg)**
Acyl ghrelin	606,100 ± 30,826	4,350 ± 1,301	UN	UN
Desacyl ghrelin	649,767 ± 64,027	4,587 ± 1,385	UN	UN
Total ghrelin	1,255,867 ± 67,509	8,937 ± 2,312	UN	UN
Obestatin	1,632 ± 57	2,600 ± 1,293	UN	710 ± 328^*^

*Acyl ghrelin, desacyl ghrelin, and obestatin immunoreactivities measured using selective assays are detected in the stomach and duodenum of ghrl+/+ mice (n = 6) but are undetectable in ghrl−/− mice (n = 6). Limits of detection are 10 pg for AG and DAG and 200 pg for obestatin. Data are expressed as Mean ± SEM. UN: Under detection limit*.

* *Immunoreactivity is unspecific*.

Ghrelin and obestatin were detected by mass spectrometry in both *ghrl*+/+ mice stomach and duodenum, only in 60% acetonitrile fractions (Table 3). Thus LC-MS profiles of SRM analyses related to preproghrelin-derived peptides in stomach and duodenum were restricted to 60% acetonitrile fractions from *ghrl*+/+ and *ghrl*−/− mice (Figure 1). The antibody used in the obestatin immunoassay may detect other forms of obestatin, such as a truncated peptide, des(1–10)-obestatin, and a modified form of obestatin with additional glycine at the C-terminal position, obestatin-Gly. Thus, the presence of des(1–10)-obestatin and obestatin-Gly was also assayed by SRM in order to evaluate a possible antibody cross-reactivity. To develop a specific and sensitive detection method for the peptides of interest, a mix of AG, DAG, obestatin, Des(1–10)-obestatin and obestatin-Gly standards (100 fmol of each) was used to calibrate the SRM method before each analysis. Blanks were run to assure the absence of contaminations from standards before the analysis of samples. Comparison between the chromatographic profiles of SRM analyses related to the fractions eluted at 60% ACN of the stomach and duodenum tissues were compared between *ghrl*+/+ and *ghrl*−/− mice (Figures 1B–F). As further described below, only native obestatin-NH2 was present in the extracts analyzed (Table 3).

**Table 3:** **SRM detection of preproghrelin-derived peptides in the different chromatographic fractions (30, 40, and 60% Acetonitrile) in the stomach and duodenum of ***ghrl***+/+ mice**.

**Tissue**	**LC Fraction (%age acetonitrile)**	**Acyl ghrelin**	**Desacyl ghrelin**	**Obestatin-NH2**	**Obestatin-Gly**	**des(1–10) -obestatin**
Stomach	30	−	+	−	−	−
	40	+	−	−	−	−
	60	+	+	+	−	−
Duodenum	30	−	−	−	−	−
	40	−	−	−	−	−
	60	+	−	+	−	−

*Acyl ghrelin and obestatin-NH_2_ were detected in the stomach and duodenum whereas, desacyl ghrelin was only detected in the stomach LC fractions of ghrl+/+ mice. MS-MS analyses further confirmed that obestatin-immunoreactivity in tissues is specific to the amidated peptide but not to obestatin-Gly nor des(1–10)-obestatin*.

The three preproghrelin-derived peptides were present in stomach LC fractions of *ghrl*+/+ mice. In duodenum LC fractions of *ghrl*+/+ mice, AG, and obestatin were present while DAG was absent. As shown on Table 3, and Figure 1, SRM analyses further confirmed that obestatin-immunoreactivity in stomach and duodenum corresponded to the amidated (Figure 1D) form of the peptide but not to obestatin-Gly (Figure 1E) nor Des(1–10)-obestatin (Figure 1F). No MS signal for any preproghrelin-derived peptides was detected in stomach or duodenum of *ghrl*−/− mice. This further validated the specificity of the spectrometric signal detected (Figures 1B–F). In stomach and duodenum extracts from *ghrl*+/+ and *ghrl*−/− mice, extracted ion chromatograms related to DAG transitions (Figure 1C) presented two peaks instead of one, respectively associated to DAG retention time (21 min) and AG retention time (29 min). The “in source” neutral loss of the acyl group of AG converted partially AG to DAG after LC separation, so that DAG SRM signature can also be detected at AG retention time. On the opposite, when DAG and AG standards are analyzed separately (Figure 1A) no signals are detected at DAG retention time with AG transitions.

## Discussion

Using a combination of selective immunoassays and highly sensitive mass spectrometry, we validate the presence of a specific immunoreactivity signal for amidated obestatin in protein extracts from stomach and duodenum and further characterize the ratio of the different preproghrelin-derived peptides in mouse gastrointestinal tract. Specific presence of each preproghrelin-derived peptide was validated by the lack of signal in preproghrelin deficient mice using both immunodetection and MS.

Until now, very few studies characterized all preproghrelin-derived peptides in murine tissues. This can be explained by the lack of sensitive and specific methods to simultaneously detect the three preproghrelin-derived peptides in a given biological sample. Moreover, their relative proportions in tissues and plasma remained unclear. Previous studies using competitive immunoassays reported equimolar ratios of acyl ghrelin and obestatin in rat plasma (Zhang et al., 2005; Zizzari et al., 2007) consistent with a model previewing both peptides obtained by the processing of the same prohormone, while ratios of 2:1–4:1 were reported in humans (Germain et al., 2009, 2010). The reason for such discrepancy is unclear but this could evoke alternative splicing and/or different processing mechanism in rodents and humans. Although one study demonstrated that obestatin is produced in gastrointestinal tract in humans (Grönberg et al., 2008), two other studies failed to identify significant amounts of obestatin in rat plasma or stomach by RIA coupled to HPLC (Bang et al., 2007; Mondal et al., 2008) in contrast with the original data from Zhang and collaborators in the rat (Zhang et al., 2005). Moreover, in the study by Mondal et al., the ratio of obestatin/ghrelin in gastric fundus of rats was 0.004%, which is far less than what is expected in plasma.

These inconsistent results raise many interrogations regarding the exact obestatin site of production, the abundance of the peptide, and the specificity of the signal measured, as well as specific differences between humans and rodents. To gain more knowledge on obestatin, we explored its presence in different murine tissues, including the gastrointestinal tract using immunological detection in association with MS analyses in order to identify the positive immune signals. Our data confirm that both forms of ghrelin as well as obestatin are produced in majority in the gastrointestinal tract in mice.

We show that in stomach, DAG represents 50% of total ghrelin production while in plasma, it accounts for about 60% of total preproghrelin-derived peptides and nearly 80% of total ghrelin. The latter result is in accordance with previous studies using competitive immunoassays which demonstrated that DAG accounts for 80–90% of total circulating ghrelin (Hosoda et al., 2000). Moreover, we find that while plasma obestatin levels represent about 30% of all circulating preproghrelin-derived peptides, obestatin is 500–1,000 times less abundant than total ghrelin in stomach.

Although we find equimolar concentrations of total ghrelin and obestatin in mouse plasma, the amount of obestatin in tissues is negligible compared to those of ghrelin. Several hypotheses may explain this observation. First of all, conditions of sampling, processing and storage may be inadequate to preserve immunoreactive obestatin. Furthermore, a low processing rate of obestatin from preproghrelin in the stomach cannot be excluded. Finally, the existence of different transcripts arising from the preproghrelin gene in a tissue-specific manner (Seim et al., 2009), including a human transcript that encodes obestatin but not ghrelin, also suggests that obestatin transcripts may be produced independently of ghrelin.

The specificity of the immunoreactive detection for all three preproghrelin-derived peptides in both stomach and duodenum was assessed by MS and further validated by the absence of signal in preproghrelin deficient mice (Hassouna et al., 2014). Residual immunoreactivity for obestatin was detected in duodenum of two out of six preproghrelin deficient mice. This is the result of an artifact as no MS signal confirmed that it was actually obestatin. Furthermore, we also confirmed by MS that the immunoreactivity detected in tissues was specific of the amidated form of obestatin. No MS signal for all preproghrelin-derived peptides was present in stomach or duodenum of *ghrl*−/− mice, confirming specificity of the assay.

In this study, we used a very sensitive mass spectrometer system. Indeed detection limits is estimated to be 10–50 fmol for AG and DAG and 1–5 fmol for Obestatin-NH_2_ and its derivatives, allowing to detect very small amounts of the peptides. The specificity was verified by determining the transitions for each peptide and analyzing ions fragments. In the original study by Zhang et al. (2005), obestatin was extracted from rat stomach but its relative abundance as compared to ghrelin was not discussed. As far as we know, this is the first attempt to identify and quantify these peptides in mouse tissues and to validate the existence of a mature form of obestatin (Obestatin-NH2) in mouse gastrointestinal tract.

In conclusion, both forms of ghrelin and obestatin can be detected with very selective immunoassays coupled with MS in gastrointestinal tract in mice. In this tissue, obestatin appears to be far less abundant than AG or DAG. This could be the result of either a lower processing rate of proghrelin into mature obestatin in gastrointestinal tissues or degradation of the peptide during the different extraction/purification procedures. Whether the main source of obestatin production and/or processing is outside the gastrointestinal tract has to be further investigated.

## Author contributions

RH: contributed to the conception and design of the work, performed experiments and analyses of data, participated to manuscript redaction. DG: contributed to reagents/materials/analysis tools, performed experiments, and analyses of data, participated to manuscript redaction. GC: contributed to the acquisition and analyses of MS data and participated to manuscript redaction. JL: contributed to the experiments and acquisition of data. OF: contributed to the experiments. CT: contributed to reagents/materials/analysis tools and revised the manuscript. JV: contributed to reagents/materials/analysis tools and revised the manuscript. JE: contributed to the conception of the work and revised the manuscript. VT: contributed to the conception and design of the work, analyses, and interpretation of data, manuscript redaction.

## Funding

This work was supported by an Agence Nationale de la Recherche (ANR) Jeunes Chercheuses Jeunes Chercheurs ANR-12-JSV1-0013-01 grant to VT, University Paris Descartes Sorbonne Paris Cité, Institut National de la Santé et de la Recherche Médicale (INSERM) and Ecole Supérieure de Physique et de Chimie Industrielles (ESPCI) Paris.

### Conflict of interest statement

The authors declare that the research was conducted in the absence of any commercial or financial relationships that could be construed as a potential conflict of interest. The reviewer SF and handling Editor declared their shared affiliation, and the handling Editor states that the process nevertheless met the standards of a fair and objective review.

## References

[B1] Bang A. S. Soule S. G. Yandle T. G. Richards A. M. Pemberton C. J. (2007). Characterisation of proghrelin peptides in mammalian tissue and plasma. J. Endocrinol. 192, 313–323. 10.1677/JOE-06-0021 17283231

[B2] Bresciani E. Rapetti D. Dona F. Bulgarelli I. Tamiazzo L. Locatelli V.. (2006). Obestatin inhibits feeding but does not modulate GH and corticosterone secretion in the rat. J. Endocrinol. Invest. 29, RC16–RC18. 10.1007/BF03344175 17033254

[B3] Delhanty P. J. Neggers S. J. van der Lely A. J. (2013). Des-acyl ghrelin: a metabolically active peptide. Endocr. Dev. 25, 112–121. 10.1159/000346059 23652397

[B4] Gargantini E. Grande C. Trovato L. Ghigo E. Granata R. (2013). The role of obestatin in glucose and lipid metabolism. Horm. Metab. Res. 45, 1002–1008. 10.1055/s-0033-1351325 23950037

[B5] Germain N. Galusca B. Grouselle D. Frere D. Billard S. Epelbaum J.. (2010). Ghrelin and obestatin circadian levels differentiate bingeing-purging from restrictive anorexia nervosa. J. Clin. Endocrinol. Metab. 95, 3057–3062. 10.1210/jc.2009-2196 20339027

[B6] Germain N. Galusca B. Grouselle D. Frere D. Tolle V. Zizzari P.. (2009). Ghrelin/obestatin ratio in two populations with low bodyweight: constitutional thinness and anorexia nervosa. Psychoneuroendocrinology 34, 413–419. 10.1016/j.psyneuen.2008.10.001 18995969

[B7] Granata R. Settanni F. Gallo D. Trovato L. Biancone L. Cantaluppi V.. (2008). Obestatin promotes survival of pancreatic beta-cells and human islets and induces expression of genes involved in the regulation of beta-cell mass and function. Diabetes 57, 967–979. 10.2337/db07-1104 18162507

[B8] Grönberg M. Tsolakis A. V. Magnusson L. Janson E. T. Saras J. (2008). Distribution of obestatin and ghrelin in human tissues: immunoreactive cells in the gastrointestinal tract, pancreas, and mammary glands. J. Histochem. Cytochem. 56, 793–801. 10.1369/jhc.2008.951145 18474938 PMC2516956

[B9] Hassouna R. Zizzari P. Tomasetto C. Veldhuis J. D. Fiquet O. Labarthe A.. (2014). An early reduction in GH peak amplitude in preproghrelin-deficient male mice has a minor impact on linear growth. Endocrinology 155, 3561–3571. 10.1210/en.2014-1126 24949662

[B10] Hassouna R. Zizzari P. Viltart O. Yang S. K. Gardette R. Videau C.. (2012). A natural variant of obestatin, Q90L, inhibits ghrelin's action on food intake and GH secretion and targets NPY and GHRH neurons in mice. PLoS ONE 7:e51135. 10.1371/journal.pone.0051135 23251435 PMC3519497

[B11] Hosoda H. Kojima M. Matsuo H. Kangawa K. (2000). Ghrelin and des-acyl ghrelin: two major forms of rat ghrelin peptide in gastrointestinal tissue. Biochem. Biophys. Res. Commun. 279, 909–913. 10.1006/bbrc.2000.4039 11162448

[B12] Howard A. D. Feighner S. D. Cully D. F. Arena J. P. Liberator P. A. Rosenblum C. I.. (1996). A receptor in pituitary and hypothalamus that functions in growth hormone release. Science 273, 974–977. 10.1126/science.273.5277.974 8688086

[B13] Kojima M. Hosoda H. Date Y. Nakazato M. Matsuo H. Kangawa K. (1999). Ghrelin is a growth-hormone-releasing acylated peptide from stomach. Nature 402, 656–660. 10.1038/45230 10604470

[B14] Lauwers E. Landuyt B. Arckens L. Schoofs L. Luyten W. (2006). Obestatin does not activate orphan G protein-coupled receptor GPR39. Biochem. Biophys. Res. Commun. 351, 21–25. 10.1016/j.bbrc.2006.09.141 17054911

[B15] McKee K. K. Tan C. P. Palyha O. C. Liu J. Feighner S. D. Hreniuk D. L.. (1997). Cloning and characterization of two human G protein-coupled receptor genes (GPR38 and GPR39) related to the growth hormone secretagogue and neurotensin receptors. Genomics 46, 426–434. 10.1006/geno.1997.5069 9441746

[B16] Mondal M. S. Toshinai K. Ueno H. Koshinaka K. Nakazato M. (2008). Characterization of obestatin in rat and human stomach and plasma, and its lack of acute effect on feeding behavior in rodents. J. Endocrinol. 198, 339–346. 10.1677/JOE-08-0082 18480381

[B17] Seim I. Herington A. C. Chopin L. K. (2009). New insights into the molecular complexity of the ghrelin gene locus. Cytokine Growth Factor Rev. 20, 297–304. 10.1016/j.cytogfr.2009.07.006 19665916

[B18] Seoane L. M. Al-Massadi O. Pazos Y. Pagotto U. Casanueva F. F. (2006). Central obestatin administration does not modify either spontaneous or ghrelin-induced food intake in rats. J. Endocrinol. Invest. 29, RC13–RC15. 10.1007/BF03344174 17033253

[B19] Tolle V. Bassant M.-H. Zizzari P. Poindessous-Jazat F. Tomasetto C. Epelbaum J.. (2002). Ultradian rhythmicity of ghrelin secretion in relation with GH, feeding behavior, and sleep-wake patterns in rats. Endocrinology 143, 1353–1361. 10.1210/endo.143.4.8712 11897692

[B20] Tolle V. Zizzari P. Tomasetto C. Rio M. C. Epelbaum J. Bluet-Pajot M. T. (2001). *In vivo* and *in vitro* effects of ghrelin/motilin-related peptide on growth hormone secretion in the rat. Neuroendocrinology 73, 54–61. 10.1159/000054620 11174017

[B21] Tomasetto C. Karam S. M. Ribieras S. Masson R. Lefèbvre O. Staub A.. (2000). Identification and characterization of a novel gastric peptide hormone: the motilin-related peptide. Gastroenterology 119, 395–405. 10.1053/gast.2000.9371 10930375

[B22] Yamamoto I. Numao M. Sakaguchi Y. Tsushima N. Tanaka M. (2007). Molecular characterization of sequence and expression of chicken GPR39. Gen. Comp. Endocrinol. 151, 128–134. 10.1016/j.ygcen.2006.12.002 17239877

[B23] Yang J. Brown M. S. Liang G. Grishin N. V. Goldstein J. L. (2008). Identification of the acyltransferase that octanoylates ghrelin, an appetite-stimulating peptide hormone. Cell 132, 387–396. 10.1016/j.cell.2008.01.017 18267071

[B24] Zhang J. V. Ren P.-G. Avsian-Kretchmer O. Luo C.-W. Rauch R. Klein C.. (2005). Obestatin, a peptide encoded by the ghrelin gene, opposes ghrelin's effects on food intake. Science 310, 996–999. 10.1126/science.1117255 16284174

[B25] Zizzari P. Longchamps R. Epelbaum J. Bluet-Pajot M. T. (2007). Obestatin partially affects ghrelin stimulation of food intake and growth hormone secretion in rodents. Endocrinology 148, 1648–1653. 10.1210/en.2006-1231 17204551 PMC1890395

