# Supplementary material for: Combination of Selective Immunoassays and Mass Spectrometry to Characterize Preproghrelin-Derived Peptides in Mouse Tissues
Source: Front Neurosci. 2017 Apr 20;11:211. doi: 10.3389/fnins.2017.00211 (PMC5397466; doi:10.3389/fnins.2017.00211)
Supplement: Supplementary file 1 [file DataSheet1.DOCX]

**Supplementary Material:**

**Combination of selective immunoassays and mass spectrometry to characterize preproghrelin-derived peptides in mouse tissues**

Rim Hassouna^1, 2§^, Dominique Grouselle^1^^§^, Giovanni Chiappetta^3^, Joanna Lipecka^1^, Oriane Fiquet^1^, Catherine Tomasetto^4^, Joëlle Vinh^3^, Jacques Epelbaum^1, 5^, Virginie Tolle^1*^

^1^UMR-S 894 INSERM, Centre de Psychiatrie et Neurosciences, Université Paris Descartes, Sorbonne Paris Cité, Paris, France

^2^Naomi Berrie Diabetes Center, Department of Pediatrics, Columbia University Medical Center, New York, NY, USA.

^3^USR-3149 ESPCI, Spectrométrie de Masse Biologique et Protéomique, PSL Research University, Paris, France

^4^UMR-7104 CNRS/U596 INSERM, Institut de génétique et de biologie moléculaire et cellulaire (IGBMC), Université de Strasbourg, Illkirch, France.

^5^UMR 7179 CNRS, MNHN, Adaptive mechanism and Evolution (MECADEV), Brunoy France

^§^*Both authors participated equally to this work*

*All correspondence and reprint requests should be addressed to:

Virginie Tolle

Phone : +33 (0) 1 40 78 92 75, Fax : +33 (0) 1 45 80 72 93

e-mail : virginie.tolle@inserm.fr

**
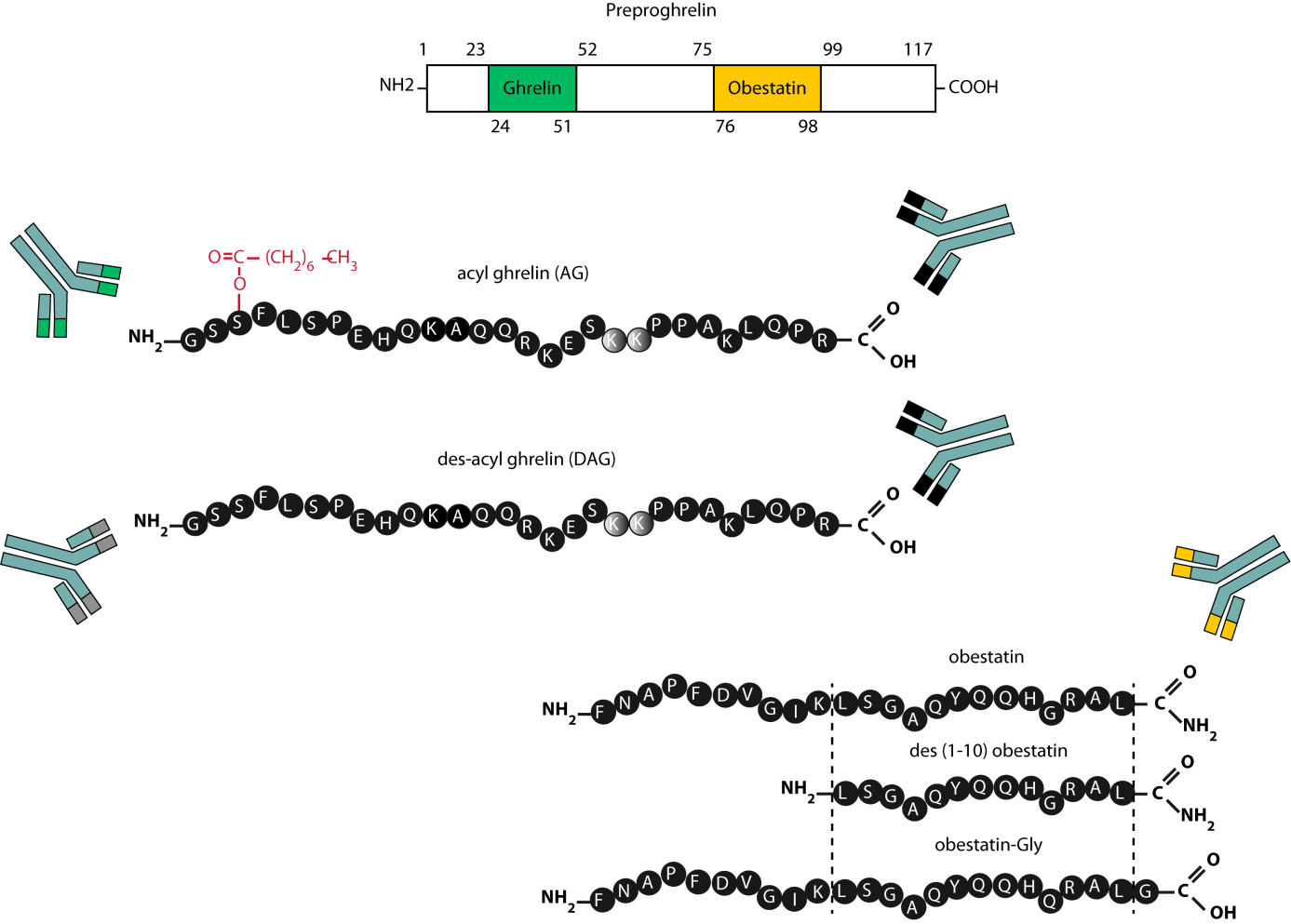
**

**Figure S1. Structure of the preproghrelin prohormone, preproghrelin-derived peptides sequences and specific antibodies to detect peptides immunoreactivities.** The preproghrelin is a 117 amino-acid prohormone that encodes several preproghrelin-derived peptides obtained from post-translational processing. Acyl ghrelin (AG), desacyl ghrelin (DAG) and obestatin immunoreactivities are measured using selective “sandwich” (for AG and DAG) or “competitive” (for obestatin) assays. The revelation antibodies are raised against the N-terminal acylated portion for AG, the N-terminal non acylated portion of DAG and the amidated C-terminal portion for obestatin. The antibodies raised against Obestatin-NH2 cross-react with Des(1-10)-Obestatin and Obestatin-Gly.

**Table S1. Sequences and specific transitions of the different preproghrelin-derived peptides.** Standard peptides used in liquid chromatography (LC): rat/mouse acyl ghrelin (AG) and desacyl ghrelin (DAG), rat/mouse amidated obestatin (Obestatin-NH_2_), Des(1-10)-Obestatin and Obestatin-Gly.

| **Peptides** | **Sequence** | **Transitions** |
| --- | --- | --- |
| Rat/mouse acyl ghrelin (AG) | Gly-Ser-Ser(O-n-octanoyl)-Phe-Leu-Ser-Pro-Glu-His-Gln-Lys-Ala-Gln-Gln-Arg-Lys-Glu-Ser-Lys-Lys-Pro-Pro-Ala-Lys-Leu-Gln-Pro-Arg | 553.1 (precursor MH_6_^6+^) → 513.3 (y_4_); 641.4 (y_5_); 712.4;(y_6_); 809.5 (y_7_) 906.5(y_8_) |
| Rat/mouse desacyl ghrelin (DAG) | Gly-Ser-Ser-Phe-Leu-Ser-Pro-Glu-His-Gln-Lys-Ala-Gln-Gln-Arg-Lys-Glu-Ser-Lys-Lys-Pro-Pro-Ala-Lys-Leu-Gln-Pro-Arg | 532.1 (precursor MH_6_^6+^) → 513.3 (y_4_); 641.4 (y_5_); 712.4; (y_6_); 809.5 (y_7_); 906.5(y_8_ |
| Rat/mouse amidated obestatin (Obestatin-NH_2_) | Phe-Asn-Ala-Pro-Phe-Asp-Val-Gly-Ile-Lys-Leu-Ser-Gly-Ala-Gln-Tyr-Gln-Gln-His-Gly-Arg-Ala-Leu-NH₂ | 630.8 (precursor MH_5_^5+^) → 262.1(b_2_); 416.2 (y_4_); 553.3 (y_5_); 681.4;(y_6_); 972.5 (y_8_) |
| Rat/mouse Des(1-10)-Obestatin | Leu-Ser-Gly-Ala-Gln-Tyr-Gln-Gln-His-Gly-Arg-Ala-Leu-NH₂ | 858.8 (precursor MH_3_^3+^) → 262.1 (b_2_); 333.2 (b_3_); 473.2 (y_5_); 610.3 (y_6_) 1029.5(y_9_) |
| Rat/mouse Obestatin-Gly | Phe-Asn-Ala-Pro-Phe-Asp-Val-Gly-Ile-Lys-Leu-Ser-Gly-Ala-Gln-Tyr-Gln-Gln-His-Gly-Arg-Ala-Leu-Gly | 476.9 (precursor MH_3_^3+^) → 201.1 (b_2_); 553.2 (y_5_); 681.3;(y_6_); 809.5 (y_7_); 972.5(y_8_) |
